# Antibiotic resistance genes detected in lichens: insights from *Cladonia stellaris*

**DOI:** 10.1093/aob/mcaf231

**Published:** 2025-09-22

**Authors:** Marta Alonso-García, Paul B L George, Samantha Leclerc, Marc Veillette, Caroline Duchaine, Juan Carlos Villarreal A

**Affiliations:** Institut de Biologie Intégrative et des Systèmes (IBIS), Université Laval, Québec G1V 0A6, Canada; Département de Biologie, Université Laval, Québec G1V 0A6, Canada; Departamento de Biología Vegetal, Universidad de Murcia, Murcia 30100, Spain; Département de Biochimie, Microbiologie et de bio-Informatique, Université Laval, Québec G1V 0A6, Canada; Institut Universitaire de Cardiologie et de Pneumologie de Québec, Université Laval, Québec G1V 4G5, Canada; Département de Biochimie, Microbiologie et de bio-Informatique, Université Laval, Québec G1V 0A6, Canada; Institut Universitaire de Cardiologie et de Pneumologie de Québec, Université Laval, Québec G1V 4G5, Canada; Institut Universitaire de Cardiologie et de Pneumologie de Québec, Université Laval, Québec G1V 4G5, Canada; Département de Biochimie, Microbiologie et de bio-Informatique, Université Laval, Québec G1V 0A6, Canada; Institut Universitaire de Cardiologie et de Pneumologie de Québec, Université Laval, Québec G1V 4G5, Canada; Institut de Biologie Intégrative et des Systèmes (IBIS), Université Laval, Québec G1V 0A6, Canada; Département de Biologie, Université Laval, Québec G1V 0A6, Canada; Royal Botanic Garden Edinburgh, 20A Inverleith Row, Edinburgh EH3 5LR, UK

**Keywords:** Antibiotic resistance genes, beta-lactamase, bioaerosols, boreal ecosystems, *Cladonia stellaris*, coevolution, lichen microbiome, lichen woodlands, mobile genetic elements, quinolone resistance

## Abstract

**Background and Aims:**

Antibiotics are natural compounds produced by microorganisms that have long existed in ecosystems. However, the widespread clinical and agricultural use of antibiotics has intensified selective pressures on bacteria, leading to the proliferation of antibiotic resistance genes (ARGs). The increasing prevalence of these genetic elements in clinical and environmental settings now poses a major global health threat. While ARGs are well documented in anthropogenically influenced environments, their distribution and origins in remote ecosystems, such as boreal forests, remain poorly understood. Here, we investigate the occurrence, diversity and potential origins of ARGs in the boreal lichen *Cladonia stellaris*.

**Methods:**

We conducted the first targeted assessment of ARGs in lichens by analysing 42 *C. stellaris* samples from northern and southern lichen woodlands in eastern Canada. Using high-throughput quantitative PCR, we screened for 33 ARGs and three mobile genetic elements (MGEs), quantifying their relative abundance. Bacterial community composition was characterized via 16S rRNA gene sequencing. Statistical analyses evaluated geographical patterns, co-occurrence between ARGs and bacterial taxa, and the influence of latitude on ARG distribution.

**Key Results:**

Ten ARGs conferring resistance to four antibiotic classes (aminoglycosides, beta-lactams, quinolones and sulfonamides), along with one MGE, were detected. The ARGs *blaCTX-M-1*, *qnrB* and *qepA* were highly prevalent, with *qepA* often surpassing 16S rRNA gene abundance. Only *qnrB* showed significantly higher abundance in southern samples. Latitude significantly influenced ARG profiles, whereas bacterial community composition did not.

**Conclusions:**

Our findings demonstrate that *C. stellaris* harbours diverse ARGs in remote boreal ecosystems with limited anthropogenic influence. Proposed explanations for ARG presence include long-distance dispersal via bioaerosols and endogenous development within lichen microbiomes, yet these remain speculative. Future work incorporating bacterial isolation, whole-genome sequencing, metatranscriptomics, air sampling and metabolomic profiling is necessary to unravel the ecology and evolution of ARGs in natural habitats.

## INTRODUCTION

Antibiotics are a class of secondary metabolites naturally produced by microorganisms, such as bacteria or fungi, or chemically synthesized analogous compounds (Demain and Sanchez, 2009). In nature, they serve various ecological roles, acting as signalling molecules and defence mechanisms. They provide producing organisms with a competitive advantage by inhibiting the growth of rival microbes, thus securing resources and space (Waksman and Woodruff, 1940). According to the arms-shield hypothesis, the production of antibiotics and the development of resistance to them is an ongoing evolutionary battle (Han *et al*., 2022). Some microorganisms have evolved the ability to produce antibiotics as a means of defence, while their competitors have developed resistance mechanisms through natural selection, enabling them to survive antibiotic exposure. This resistance capability is conferred by antibiotic resistance genes (ARGs), which are segments of DNA encoding proteins that enable bacteria to survive exposure to antibiotics. ARGs can develop intrinsically within the bacterial genome or acquired through horizontal gene transfer (HGT) (Thomas and Nielsen, 2005; Barlow, 2009) from other bacteria via mobile genetic elements (MGEs) often associated with plasmids (Partridge *et al*., 2018; Razavi *et al*., 2020; Ni *et al*., 2023), or via transduction from bacteriophages (Balcazar, 2014; Penadés *et al*., 2015). Their existence in the environment pre-dates the anthropogenic use of antibiotics (Wright, 2007; Martínez, 2008) with evolutionary evidence tracing their development from thousands (D’Costa *et al*., 2011) to billions of years (Hall and Barlow, 2004).

Since the 1940s, when antibiotics began to be used in medicine, their application has expanded to sectors such as agriculture (e.g. Anderson and Gottlieb, 1952; McManus *et al*., 2002), aquaculture (e.g. Cabello, 2006; Chen *et al*., 2020) and animal husbandry (e.g. Hong *et al*., 2013; Busch *et al*., 2020; Karwowska, 2024). Such extensive, and at times inappropriate, use of antibiotics has led to the proliferation of ARGs and ARG-carrying bacteria in both natural and anthropogenic ecosystems. The World Health Organization has identified antibiotic resistance as one of the top public health threats of the 21st century. In 2019 alone, over 4.95 million deaths were linked to antibiotic resistance, with approximately 1.27 million directly attributed to infections caused by antibiotic-resistant bacteria (Murray *et al*., 2022). At the national level, antimicrobial resistance was responsible for the deaths of approximately 5400 Canadians in 2018. If current trends continue, resistance rates are projected to increase from 26 % in 2018 to 40 % and potentially up to 396 000 cumulative deaths by 2050, with economic losses ranging from CA$13 to 21 billion (Council of Canadian Academies, 2019).

The spread of ARGs in the environment occurs not only through local contamination near anthropogenic sources, such as the direct release of ARG-carrying bacteria via wastewater discharge or agricultural runoff (Almakki *et al*., 2019; Junaid *et al*., 2022), but also by atmospheric processes that enable their dispersal over much greater distances. Bioaerosols, which are airborne particles of biological origin encompassing bacteria, viruses and fungi, can carry ARGs over distances ranging from metres to hundreds of kilometres (Brunet *et al*., 2017; Griffin *et al*., 2017). Bioaerosols are transported by atmospheric currents (Jin *et al*., 2022; Wang *et al*., 2022), before settling out of the air through dry deposition (gravity-driven settling onto surfaces) or precipitation into terrestrial or aquatic environments (Di Cesare *et al*., 2017; O’Malley *et al*., 2023). Such deposition can reach even remote ecosystems, which thus become valuable settings for investigating the potential long-distance dispersal of ARGs. In this context, lichen woodlands (LWs) offer a suitable system, as their open canopies provide little physical obstruction to airborne particles, allowing microorganisms suspended in the atmosphere to settle directly onto the ground layer. LWs cover approximately 2 million km^2^ of Canada, including nearly 300 000 km^2^ in the province of Quebec (Payette and Delwaide, 2018). They are situated north of the closed-crown forest zone and south of the forest–tundra zone, between 52.00°N and 55.00°N, in remote regions characterized by low human population density and limited anthropogenic activities (Bhiry *et al*., 2011). An exception to the typical distribution of LWs occurs in Parc National des Grands-Jardins, Quebec (PNGJ; 47.68°N, 70.85°W), where a LW is found 500 km south of its usual range (Jasinski and Payette, 2005).

Within LWs, the ground layer is dominated by a continuous and exposed cover of lichens (Payette *et al*., 2001; Girard *et al*., 2017), with *Cladonia stellaris* (Opiz) Pouzar & Vězda being the dominant and most representative species. Lichens are well known for their ability to absorb and accumulate substances from their surrounding environment, including airborne particles. Beyond their role as passive accumulators, lichens also produce a variety of antimicrobial compounds (Burkholder *et al*., 1944; Yilmaz *et al*., 2004; Rankovič *et al*., 2010; Shrestha and St. Clair, 2013), which exert selective pressure on their associated bacterial communities (Grube *et al*., 2009; Grimm *et al*., 2021), and may promote the development of resistance, as proposed for the lichen species *Rhizocarpon geographicum* (Miral *et al*., 2022). In this context, LWs provide a unique system to investigate ARG dynamics, encompassing both the deposition of airborne ARGs and the potential development of endogenous resistance mechanisms within lichen-associated bacterial communities.

To investigate these patterns, we conducted the first targeted assessment of ARGs in lichen samples using high-throughput quantitative PCR (HT-qPCR). The objectives of this study are to (1) test the presence of ARGs in lichens from both northern and southern LWs, (2) quantify their abundance, (3) compare the diversity and abundance of ARGs between northern and southern LWs, and (4) explore the potential relationship (correlations) between their ARG profiles and constituent bacterial communities. To frame our investigation, we consider two potential explanations for the presence of bacteria-carrying ARGs in *C. stellaris*: first, that long-distance dispersal by bioaerosols introduces ARGs to LWs, by transporting bacteria-carrying ARGs from urban and agricultural environments where antibiotics are frequently used (George *et al*., 2022; Kormos *et al*., 2022); and second, that ARGs in lichen-associated communities may be indigenous, developing as a response to selective pressures imposed by the antimicrobial compounds produced by lichens (Francolini *et al*., 2004; Goga *et al*., 2021; Zorrilla *et al*., 2022). As this is the first exploratory assessment of ARGs in lichens, these explanations remain hypotheses that will need to be tested in future targeted studies.

## MATERIAL AND METHODS

### Study areas and sampling

**Fig. 1.**
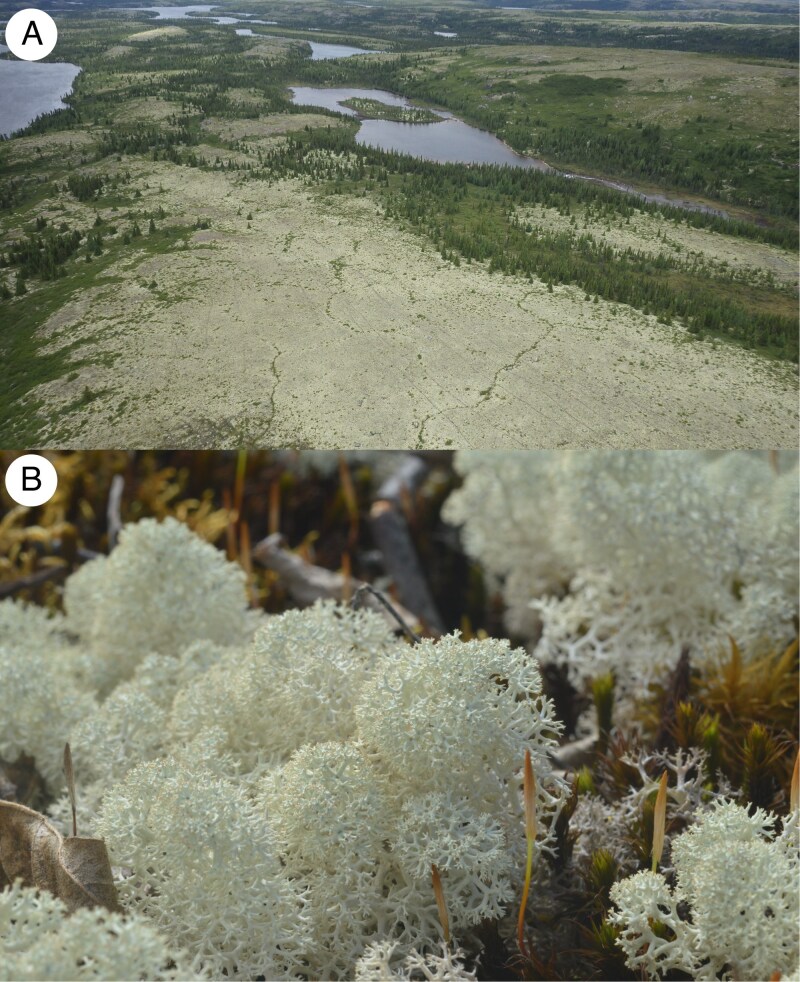
(A) Representative lichen woodland dominated by *Cladonia stellaris*. (B) Close-up of the lichen *C. stellaris*, showing the thallus morphology characteristic of this species (photo credits: A. Claude Morneau, B. Philip Bell-Doyon).

This study builds upon data previously collected by Alonso-García *et al*. (2021), and Alonso-García and Villarreal A. (2022), which investigated the factors shaping the bacterial community composition of lichens across Quebec, Canada. *Cladonia stellaris* samples were collected from two locations: the first near Kuujjuarapik–Whapmagoostui (55.28°N, −77.75°W) in the north and from PNGJ (47.68°N, −70.85°W) in the south. Figure 1 shows the LW ecosystem and a close-up of the lichen species. Kuujjuarapik–Whapmagoostui has a mean annual temperature of −3.6 °C and a mean annual precipitation of 640 mm (Prairie Climate Centre University of Winnipeg, 2022). In this region, LWs develop on sandy terraces and wind-affected areas, where *Picea glauca* (Moench) Voss and *Picea mariana* (Mill.) Britton, Sterns & Poggenb. coexist with a ground cover dominated by *C. stellaris* and ericaceous shrubs such as *Betula glandulosa* Michx., *Rhododendron groenlandicum* Retzius and *Salix glauca* L. (Bhiry *et al*., 2011). These open forests grow on acidic, nutrient-poor, well-drained soils (Payette, 1993; Payette *et al*., 2001).

The PNGJ has a mean annual temperature of 2 °C, and an annual precipitation of approximately 1200 mm (Payette *et al*., 2000; Prairie Climate Centre University of Winnipeg, 2022). The park features a mosaic of closed-crown spruce–moss forests and open-crown lichen woodlands dominated by *P. mariana*, Ericaceae species and *C. stellaris* (Jasinski and Payette, 2005), thriving on acidic, nutrient-depleted moraine-derived soils and granitic outcrops (Payette, 1992; Payette and Morneau, 1993). Although both regions are relatively remote compared to urban centres, potential sources of anthropogenic contamination exist near both study sites. In Kuujjuarapik–Whapmagoostui, there is a possibility that domestic waste, healthcare services and potential wastewater seepage may introduce antibiotics or resistant organisms into nearby ecosystems. The PNGJ has protected status, restricting human activities. However, it is located within the Charlevoix region, where agriculture and livestock farming are widespread (MRC de Charlevoix, 2022), and its proximity to the town of Baie-Saint-Paul (less than 30 km away) could expose the park to anthropogenic contaminants.

Sampling was performed in 2018 using sterilized steel forceps, targeting thalli of *C. stellaris*, which were immediately stored at −20 °C. Further methodological details are available in Alonso-García *et al*. (2021). In the present study, 42 samples were selected for analyses: 18 from seven sites around Kuujjuarapik–Whapmagoostui (northern LW) and 24 from PNGJ (southern LW) (Supplementary Data Table S1). Each sample corresponded to one individual thallus, with no technical replicates performed.

### Data processing and analysis of 16S rRNA genes

To characterize bacterial community composition, we used amplicons of the V3–V4 region of the 16S rRNA gene generated by Alonso-García and Villarreal A. (2022). These data were passed through an established DADA2 pipeline (Callahan *et al*., 2016) in R v.4.4.2 (R Core Team, 2021) for processing amplicon sequence variants (ASVs, Callahan *et al*., 2017). We transformed ASV counts to relative abundances using the phyloseq package v.1.38 (McMurdie and Holmes, 2013) for comparisons of biodiversity between northern and southern LWs. To calculate the Shannon diversity index, we first normalized the ASV table using the DESeq2 package v.1.38 (Love *et al*., 2014), applying the poscounts method. This approach estimates size factors using the median-of-ratios method while excluding zero counts, making it particularly suitable for sparse amplicon data typical of microbial community profiles. We tested for differences in bacterial alpha diversity between LWs using unpaired *t*-tests, after confirming normality and homogeneity of variances using the Shapiro–Wilk and Levene's tests, respectively. To analyse beta diversity, we built a Bray–Curtis distance matrix based on relative abundances and performed a principal coordinates analysis (PCoA). We assessed differences in bacterial community composition between LWs with a permutational multivariate analysis of variance (PERMANOVA) test, preceded by a check of the homogeneity of dispersions using the betadisper function from the vegan package v.2.6 (Oksanen *et al*., 2024). We used the Bray–Curtis distance matrix from the relative abundance data to perform distance-based redundancy analysis (db-RDA) to evaluate the influence of the latitude of LW on the bacterial community composition, fitting the model with the capscale function in the vegan package (Oksanen *et al*., 2024), with LW as the explanatory variable. The significance of the db-RDA model was assessed using permutation tests.

### SmartChip high-throughput quantitative PCR

Bacterial biomass was assessed via qPCR using the 16S rRNA marker gene. Primers and probes are as described in Bach *et al*. (2002). We conducted qPCR analyses using a Bio-Rad CFX-384 Touch™ Real-Time PCR Detection System (Bio-Rad, Montreal, Canada) under the following thermoprotocol: 95 °C for 3 min; followed by 40 cycles of 95 °C for 20 s and 62 °C for 1 min. Results were validated only if the accompanying standard curves showed efficiency values between 90 and 110 %.

We employed the SmartChip Real-Time PCR System (Takara Bio USA, Inc.) to screen for the presence of ARGs and MGEs. HT-qPCR was performed following the manufacturer's protocol. Each 250 μL reaction contained the appropriate primers for the targeted ARGs, along with the lichen DNA extracted previously in Alonso-García and Villarreal A. (2022) . The selection of ARGs was guided by the framework established by George *et al*. (2022), ensuring comparability with previous global studies. We targeted 33 ARGs and three MGEs (Table 1). We included positive controls consisting of synthetic target gene sequences at concentrations of 10^6^, 10^3^, and 10^1^ copies per μL, along with negative (no-template) controls, to verify specificity and the absence of contamination. Each sample represented a single thallus (biological replicate), and ARG detection was performed in technical triplicates. A gene was considered present if detected in at least two out of the three technical replicates. The *Ct* values from these replicates were averaged for further analysis.

**Table 1.** List of gene targets used for high-throughput quantitative PCR analyses.

Gene name	Gene type
*aac(6’)-II*, *aac(6’)-Ib*, *aac(3’)*	Aminoglycoside resistance
*blaCTX-M-1*, *blaGES*, *blaIMP*, *blaMOX/blaCMY*, *blaOXA*, *blaTEM*, *blaVEB*, *blaVIM*	Beta-lactam resistance
*mcr1*	Colistin resistance
*ermB*, *ermF*, *ermT*, *ermX*, *erm(35)*	Macrolide resistance
*qnrB*, *qepA*	Quinolone resistance
*sul1*, *sul2*	Sulfonamide resistance
*tet32*, *tetA*, *tetC*, *tetL*, *tetM*, *tetO*, *tetQ*, *tetS*, *tetW*, *tetX*	Tetracycline resistance
*vanA*, *vanB*	Vancomycin resistance
*IS26*	Mobile genetic element (transposase gene)
*tnpA*	Mobile genetic element (transposase gene)
*int1-a-marko*	Mobile genetic element (integrase gene)

The relative abundance of each ARG/MGE compared with 16S rRNA was calculated using the $2^{-\Delta Ct}$ method, where ΔCt=Ct(ARGorMGE)−Ct(16SrRNA); hereafter this is referred to as ARG relative abundance. To visualize the variation of ARGs across the lichen samples, we created a heat map using the relative abundance values. A logarithmic transformation (log_10_) was applied to the abundance values to better represent the wide range of abundances in the heat map. We compared ARG relative abundance between northern and southern LWs using exclusively ARGs with a prevalence greater than 50 % across all samples. To test for statistical differences in ARG abundance between northern and southern LWs, we performed Wilcoxon rank-sum tests with Benjamini–Hochberg (BH) correction due to a non-normal distribution of the data revealed by the Shapiro–Wilk test. Prior to testing, abundance values of zero were excluded from the dataset to avoid skewing the results. Additionally, we assessed the overall ARG abundance in each lichen sample, by summing the relative abundances of all ARGs for each sample, and we applied a Wilcoxon rank-sum test to compare it between the two LWs. We assessed the influence of the latitude of LW on the relative abundance of the 11 ARGs detected in *C. stellaris* by performing a db-RDA on the Bray–Curtis distance matrix of ARG relative abundance as described above.

### Comparison of ARGs and bacterial communities in lichens

We assessed how much of the variance in the relative abundance of ARGs could be explained by the relative abundance of bacterial families. The relative abundances of ARGs served as the response variables, while the relative abundances of bacterial families were the explanatory variables. To check for multicollinearity among the explanatory variables, we calculated Spearman's correlation coefficients. We used a threshold of 0.4 for correlations to identify and remove highly co-correlated bacterial families from the analysis. The removed families were: Bdellovibrionaceae, Sphingomonadaceae, Isosphaeraceae, SM2D12, Caulobacteraceae and Microbacteriaceae. Given that the linearity assumption between ARGs and bacterial families was not confirmed, we opted to use canonical correspondence analysis (CCA), which can handle non-linear relationships. The significance of the CCA model was tested using ANOVA with permutation.

To explore potential relationships between ARGs and associated bacterial taxa, we conducted network analyses. First, we prepared a dataset of combined ARG relative abundances with bacterial genera abundances, transformed using centred log-ratio to standardize the data. We calculated Spearman correlation coefficients (|ρ|) and corresponding *P*-values using the rcorr function from the Hmisc package in R (Harrell, 2024). We set significance thresholds at a correlation coefficient greater than 0.2 and an adjusted *P*-value below 0.05. To control for multiple testing, we applied BH correction to the *P*-values. Significant correlations were retained, and their values were used to construct the network, where nodes represent ARGs or bacterial taxa, and edges represent significant correlations between them. It is important to note that these correlations indicate co-variation patterns and do not confirm direct genetic linkage between ARGs and bacterial taxa. The network was generated using the igraph package in R (Csardi and Nepusz, 2006), with edge weights corresponding to the absolute values of the Spearman correlation coefficients. The direction of the correlation (positive or negative) was annotated, and correlation strength was categorized as weak, moderate, strong or very strong based on the absolute value of the correlation coefficient (ranges: 0.2–0.4, 0.4–0.6, 0.6–0.8 and 0.8–1.0, respectively). For further visualization and editing, we exported the network data, including node attributes and edges with adjusted *P*-values (BH correction), to a CSV file compatible with Cytoscape software v.3.10 (Shannon *et al*., 2003).

## RESULTS

### Bacterial community differs between LW

**Fig. 2.**
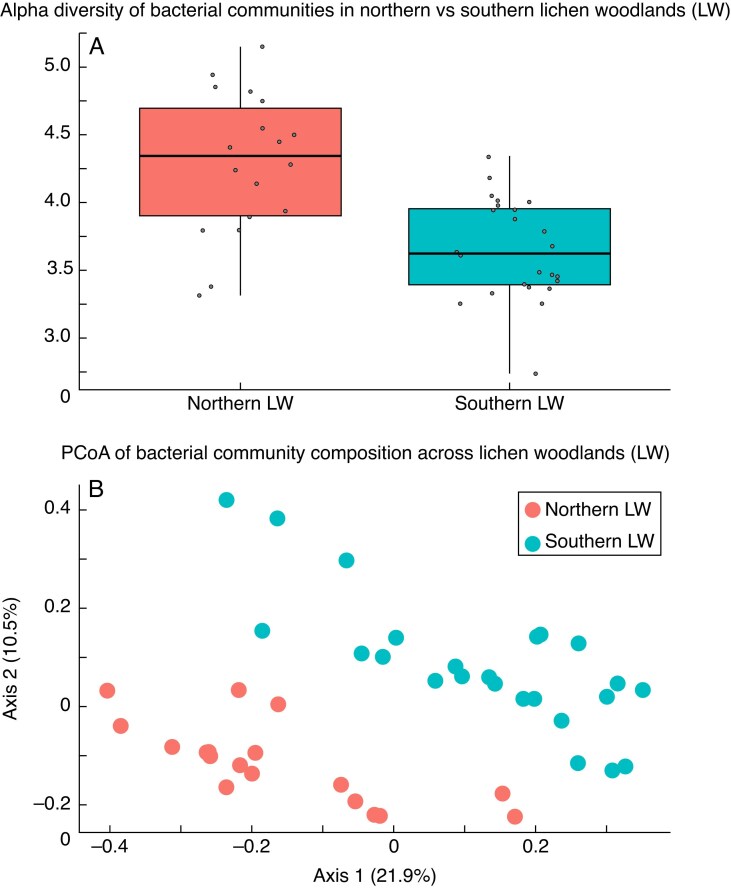
(A) Alpha diversity Shannon index of bacteria associated with *Cladonia stellaris* from northern and southern lichen woodlands (LWs). The median (horizontal line), quartiles (edges of the box) and 1.5× the interquartile range (whiskers) are displayed. Points represent individual observations. Student’s *t*-test results indicated a significant difference in alpha diversity between the two LWs (*P* < 0.05). (B) Principal coordinates analysis (PCoA) of bacteria associated with *C. stellaris* from northern and southern LWs. Samples are coloured according to LW latitude. PERMANOVA results indicated a significant difference in the bacterial community composition between the two LWs (*R* = 0.14303, *P* < 0.01).

A total of 481 unique ASVs were retained after quality and prevalence filters from 48 lichen samples, a number consistent with previous reports for lichen- and moss-associated bacterial communities using similar prevalence thresholds (Alonso-García and Villarreal A. 2022; Escolástico-Ortiz *et al*., 2023). The ASVs were assembled into ten phyla. Proteobacteria was the most abundant, accounting for 242 ASVs. The next most abundant phyla were Acidobacteriota (86 ASVs), Planctomycetota (78 ASVs) and Verrucomicrobiota (42 ASVs) (Supplementary Data Fig. S1A). Among the ASVs assigned to genera, *Tundrisphaera* (65 ASVs), *Granulicella* (41 ASVs) and LD29 (33 ASVs) were the most abundant, while genera such as *Conexibacter*, *Terriglobus* and *Novosphingobium* were represented by only one or two ASVs each (Fig. S1B, Table S2). Unpaired *t*-tests revealed that alpha diversity was higher in northern than in southern LWs (*P* = 0.0001) (Fig. 2A). The Bray–Curtis distance-based PCoA showed that bacterial communities of *C. stellaris* differ between LWs, with no overlap, with axes 1 and 2 explaining 22 and 10.5 % of the total variance, respectively (Fig. 2B). PERMANOVA results confirmed that the separation between LWs was statistically significant (*R* = 0.143, *P* = 0.001). The db-RDA model revealed that the latitude of LW explained 14.23 % of the variance in the bacterial community composition. The global permutation test confirmed that the model was statistically significant (*F* = 6.634, *P* = 0.001).

### Characterization of ARGs present in lichens

**Fig. 3.**
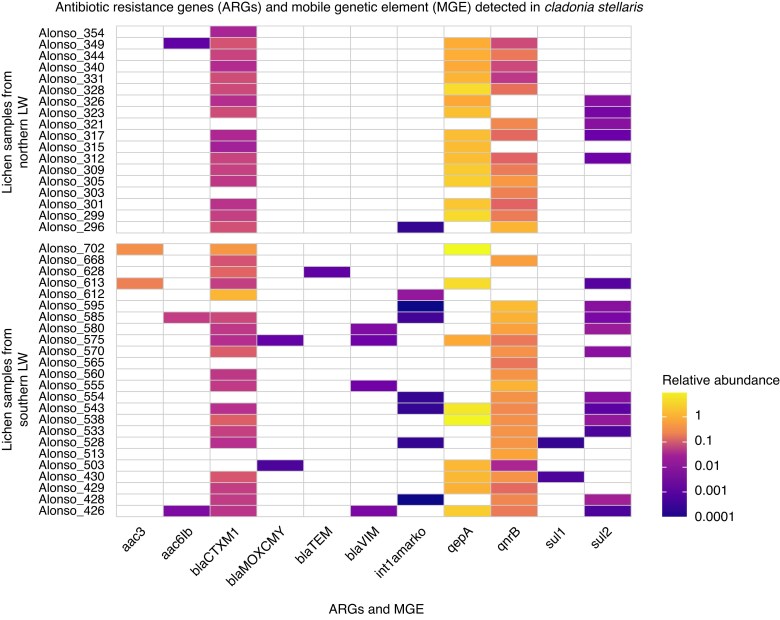
Heat map of ten antibiotic resistance genes (ARGs) and one mobile genetic element (MGE) found in *Cladonia stellaris* samples. Each tile represents the logarithmic (log_10_) abundance of a specific ARG/MGE in each lichen sample, with darker colours indicating higher abundance and white tiles indicating an absence of the gene. Samples are grouped by lichen woodland (LW) latitude, on the *y*-axis, while the ARGs/MGE are displayed on the *x*-axis.

Among the 33 ARGs and three MGEs tested, ten ARGs and one MGE were detected in *C. stellaris* samples, corresponding to resistance to aminoglycosides [*aac(6’)-Ib* and *aac(3’)*], beta-lactams (*blaCTX-M-1*, *blaMOX/CMY*, *blaTEM* and *blaVIM*), quinolones (*qepA* and *qnrB*) and sulfonamides (*sul1* and *sul2*), as well as one MGE (*int1-a-marko*). The relative abundances of the detected ARGs and MGE are shown in Fig. 3 and Supplementary Data Table S3. While most ARGs and the MGE exhibited lower abundance compared to the 16S rRNA gene, *qepA* was an exception, displaying higher relative abundance in most samples (Fig. 3).

**Fig. 4.**
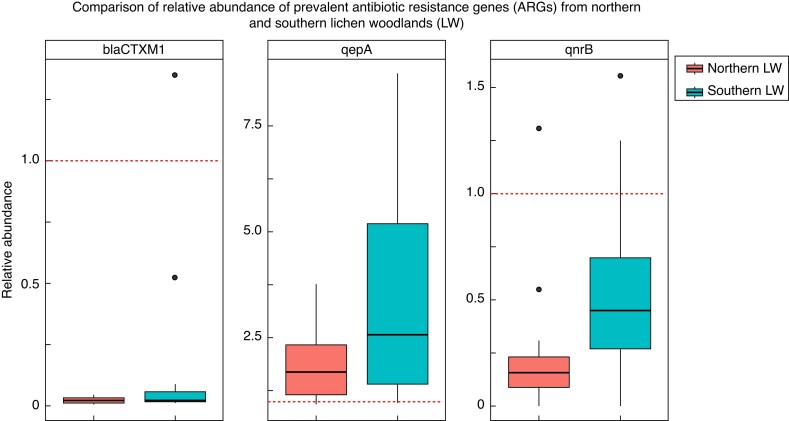
Relative abundance of three prevalent antibiotic resistance genes (ARGs) in *Cladonia stellaris* from northern and southern lichen woodlands (LWs). The median (horizontal line), quartiles (edges of the box) and 1.5× the interquartile range (whiskers) are displayed. Points represent individual observations. A dashed red horizontal line at *y* = 1 indicates the reference value, where values equal to 1 represent abundance equivalent to the 16S rRNA gene. Values greater than 1 indicate higher abundance compared to the 16S rRNA gene, while values less than 1 indicate lower abundance. Wilcoxon rank-sum results indicated significant differences in the relative abundance of *qnrB* between the northern and southern LWs (adjusted *P* = 0.0274).

The three most prevalent ARGs, *blaCTX-M-1*, *qnrB* and *qepA*, were detected in more than 50 % of the samples. Specifically, *blaCTX-M-1* was found in 35 samples (16 from the northern LW and 19 from the southern LW), *qnrB* in 34 samples (14 northern LW, 20 southern LW) and *qepA* in 23 samples (14 northern LW, nine southern LW) (Fig. 3; Supplementary Data Table S3). Comparisons of the relative abundance of these three ARGs between northern and southern LWs revealed a significant difference only for *qnrB* (adjusted *P* = 0.027), which was higher in southern LW (Fig. 4). However, when considering the total relative abundance of all detected ARGs combined, no significant differences were observed between the two regions (adjusted *P* = 0.37) (Fig. S2).

Finally, the influence of LW latitude on ARG composition was assessed using db-RDA. Latitude explained 12.61 % of the variance in the relative abundance of ARGs associated with *C. stellaris*, and the global permutation test confirmed that the model was statistically significant (*F* = 5.696, *P* = 0.005).

### Relationship between ARGs and bacterial communities in lichens

**Fig. 5.**
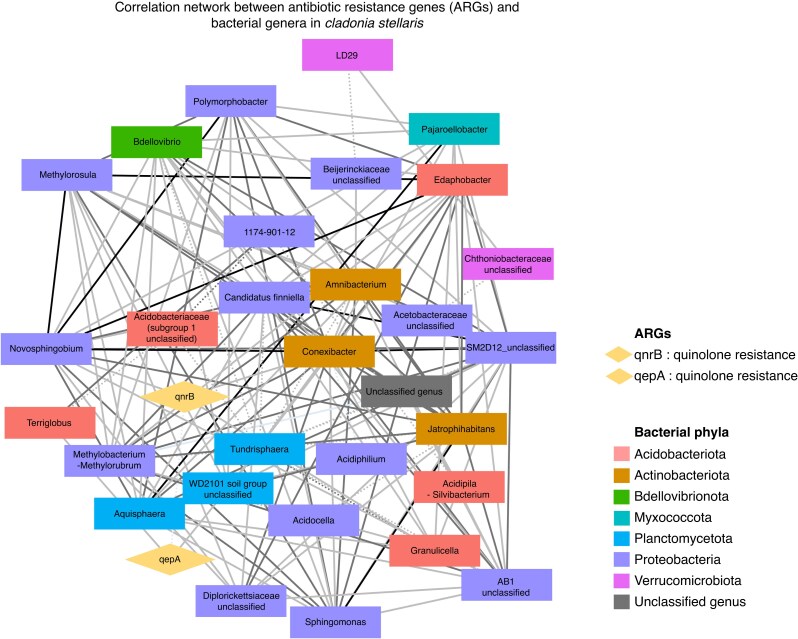
Network analysis of correlations between antibiotic resistance genes (ARGs) and bacterial genera associated with *Cladonia stellaris*. The network was constructed using Spearman correlation coefficients, displaying significant correlations (|ρ| > 0.2 and adjusted *P* < 0.05) between ARGs and bacterial genera. Nodes represent ARGs (diamonds) or bacterial genera (rectangles), with edges depicting significant correlations. Edge colour indicates correlation strength, categorized as weak (0.2 < |ρ| ≤ 0.4), moderate (0.4 < |ρ| ≤ 0.6) or strong (|ρ| > 0.6). Solid edges indicate positive correlations, and dotted edges represent negative correlations.

The CCA did not yield statistically significant results (*F* = 1.7, *P* = 0.139), indicating that the relative abundances of bacterial families did not account for a significant proportion of the variance observed in ARG relative abundances. Network analysis displayed the relationships between bacterial genera and the two quinolone ARGs (*qnrB* and *qepA*), with no connections observed for *blaCTX-M-1*. The overall network comprised 32 nodes and 147 edges, with an average of 9.19 neighbours per node, a clustering coefficient of 0.527 and a network density of 0.296 (Table 2). These metrics indicate a moderately interconnected network with a cohesive structure, where no bacterial genera or ARGs were isolated (Fig. 5).

**Table 2.** Summary of key network metrics of bacterial communities and antibiotic resistance genes (ARGs) associated with *Cladonia stellaris*.

Summary statistics	All samples
Number of nodes	32
Number of edges	147
Average number of neighbours	9.188
Network diameter	5
Network radius	3
Characteristic path length	2.109
Clustering coefficient	0.527
Network density	0.296
Network heterogeneity	0.66
Network centralization	0.372
Connected components	1

Among the genera identified, *Conexibacter* (represented by two ASVs) emerged as a key node within the bacterial community of *C. stellaris*. It exhibited the highest number of connections (20) and high centrality indices (closeness centrality = 0.67, betweenness centrality = 0.219) (Fig. 5; Supplementary Data Table S4). This genus was positively correlated with *qnrB* (|ρ| = 0.54), indicating co-variation in relative abundance across samples. *Granulicella* (41 ASVs) and *Novosphingobium* (one ASV), though differing in abundance, showed similar patterns in the network. Both genera showed moderate positive correlations with *qnrB* (|ρ| = 0.54 for *Granulicella* and |ρ| = 0.43 for *Novosphingobium*) and had relatively low betweenness centrality (0.010 for *Granulicella* and 0.027 for *Novosphingobium*). Additionally, *Granulicella* contributed eight connections to the network, indicating a moderate role in the network structure, while *Novosphingobium* had 16 connections, reflecting a stronger presence in the network (Fig. 5; Table S4). Two different genera were positively correlated with *qepA*: *Tundrisphaera* (|ρ| = 0.43) and *Terriglobus* (|ρ| = 0.45), indicating co-occurrence patterns. While *Tundrisphaera* (65 ASVs) had 11 connections, *Terriglobus* (one ASV) had only three. Both genera displayed relatively low betweenness centrality (0.089 for *Tundrisphaera* and 0.007 for *Terriglobus*) (Fig. 5; Table S4), suggesting a more localized role in linking different parts of the network. A weak but significant negative correlation was observed between *qnrB* and *qepA* (|ρ| = −0.40) (Fig. 5; Table S4), which may indicate that these two resistance genes do not co-occur frequently in the same bacterial community. However, given that *qepA* exhibits much higher relative abundance across samples compared to *qnrB*, this negative correlation may also reflect the influence of their relative abundance differences, which could affect their co-occurrence patterns.

## DISCUSSION

Bacteria associated with *C. stellaris* from southern and northern LWs in Quebec harboured ten genes conferring resistance to four classes of antibiotics: aminoglycosides [*aac(6’)-Ib* and *aac(3’)*], beta-lactams (*blaCTX-M-1*, *blaMOX/CMY*, *blaTEM* and *blaVIM*), quinolones (*qepA* and *qnrB*), and sulfonamides (*sul1* and *sul2*), as well as one MGE (*int1-a-marko*). One beta-lactam resistance gene (*blaCTX-M-1*) and two quinolones (*qepA* and *qnrB*) were detected in over 50 % of the samples, with *qepA* showing a particularly high relative abundance, surpassing the abundance of the 16S rRNA gene in most samples. Comparisons between northern and southern LWs indicated a trend toward higher ARG relative abundance in southern samples; however, only the *qnrB* gene showed a statistically significant difference. Redundancy analyses revealed that LW latitude explained almost 13 % of the variance in ARG profiles, suggesting a spatial effect. In contrast, no significant influence of bacterial family composition on ARG abundance was detected. Additionally, we identified positive correlations between *qnrB* and the bacterial genera *Connexibacter*, *Granulicella* and *Novosphingobium*, and between *qepA* and *Tundrisphaera* and *Terriglobus*. These correlations reflect co-occurrence patterns across samples, but do not provide direct evidence that these bacteria carry the ARGs. Our data confirm the presence of ARGs in *C. stellaris* across both northern and southern LWs, but the mechanisms underlying the arrival and persistence of ARGs in these ecosystems remain unclear. In the following sections, we explore two main hypotheses: (1) long-distance dispersal via bioaerosols, and (2) endogenous development driven by coevolutionary dynamics between the lichen host and its associated bacteria (arms-shields race hypothesis). These interpretations are discussed here only as possible scenarios to contextualize our findings, but the current dataset does not allow us to evaluate their likelihood. Further research will be essential to address the unresolved questions raised by our findings.

### Influence of LW latitude on bacterial community and ARG distribution

Consistent with the findings of Alonso-García and Villarreal A. (2022), our results revealed significant differences in the diversity and composition of bacterial communities associated with *C. stellaris* between northern and southern LWs, with northern LWs exhibiting higher bacterial diversity. Latitude accounted for around 14 % of the variation in the bacterial community of lichen samples and about 13 % of the variation in ARG relative abundance, suggesting that local factors specific to each LW influence both bacterial community and ARG abundance. While the impact of the local abiotic factors on bacterial community composition is well established (Cardinale *et al*., 2012; Klarenberg *et al*., 2020; Paulsen *et al*., 2024), recent studies have also demonstrated its influence in shaping ARGs abundance and distribution. Soil physicochemical properties, for example, have been shown to structure soil ARG profiles (Song *et al*., 2021; Wang *et al*., 2024). In addition to abiotic factors, biotic drivers such as microbial community composition can also influence ARG abundance (Bahram *et al*., 2018; Yan *et al*., 2021), although the strength and direction of this effect vary by habitat type. For instance, bacterial community structure influences ARG abundance in soils, whereas no such effect was observed in the phyllosphere of plants (Xiang *et al*., 2020). Our results are consistent with this complexity: while LW latitude explained a small but significant portion of the variation in ARG abundance, bacterial community composition itself had no detectable influence on abundance of those genes, suggesting that additional, as yet unidentified, factors are also driving ARG abundance in LWs. Notably, no previous studies have directly applied high-throughput qPCR to assess ARGs in lichens or other cryptogams, precluding direct comparison of our results with related systems. Future studies integrating environmental, climatic and anthropogenic data with microbial and ARG profiling, including comparative analyses across lichens, and adjacent soils, will be essential to identify the factors and mechanisms driving ARG distribution in boreal lichen woodlands.

### Frequent and abundant ARGs: beta-lactam and quinolone resistance

#### Beta-lactam resistance in lichen-associated bacteria

Beta-lactam antibiotics are among the most widely used antimicrobial agents and are naturally produced by certain microorganisms, such as the fungus *Penicillium chrysogenum* and the bacterium *Agrobacterium radiobacter* (Sykes *et al*., 1981). In this study, we detected four beta-lactamase resistance genes (*blaMOX/CMY*, *blaTEM*, *blaVIM* and *blaCTX-M-1*) in *C. stellaris* samples. Three of these genes were sporadically detected, exclusively in the southern LW (PNGJ). This sporadic detection may be related to nearby anthropogenic activities, such as agriculture and livestock farming, as beta-lactam ARGs are frequently enriched in agricultural and urban environments (Chen *et al*., 2018; Yan *et al*., 2019; Xiang *et al*., 2020). Bioaerosol transport from these areas could contribute to their subsequent deposition in PNGJ (Bai *et al*., 2022; George *et al*., 2022; Yang *et al*., 2023).

Conversely, *blaCTX-M-1* was by far the most prevalent ARG, found in 83 % of the *C. stellaris* samples. We hypothesize that *blaCTX-M-1* may be stably integrated within the lichen-associated microbiome. Although lichens are not known to produce beta-lactams, they synthesize a wide array of antimicrobial compounds (Burkholder *et al*., 1944; Aslan *et al*., 2006; Rankovič *et al*., 2010; Mitrović *et al*., 2011; Shrestha and St. Clair, 2013; Kosanić *et al*., 2014; Kosanić and Rankovic, 2015), which could exert selective pressures favouring *blaCTX-M-1* retention over evolutionary timescales. Noël *et al*. (2021) demonstrated that lichen-associated bacteria developed mechanisms to survive in the presence of antibacterial compounds produced by lichens, and Agersø *et al*. (2019) suggested that some ARGs may be intrinsic to bacteria and originated from ancient resistomes rather than recent contamination.

In our dataset, *blaCTX-M-1* did not show significant correlations with specific bacterial genera, a pattern also reported for *blaTEM* (Jang *et al*., 2022) and *aac-(6)-Ib* (Yan *et al*., 2017). A plausible explanation for this lack of association is the frequent linkage of these genes to MGEs (Cantón *et al*., 2012; Partridge *et al*., 2018; Cormier *et al*., 2022), which are known to facilitate HGT among diverse bacterial taxa. Such mechanisms may enable *blaCTX-M-1* to disseminate widely across microbial communities without producing detectable genus-level co-variation (Zhu *et al*., 2017; Li *et al*., 2023), potentially contributing to its broad occurrence across both northern and southern LWs.

#### Quinolone resistance in lichen-associated bacteria

Quinolones are synthetic antibiotics introduced in the 1960s, with widespread use established by the 1980s (Hooper and Jacoby, 2015). They are extensively employed in human and veterinary medicine, as well as in food production (World Health Organization, 2018), contributing to growing contamination of natural environments (Cattoir *et al*., 2008; Ben *et al*., 2019; Zhai *et al*., 2024). In this study, we detected two quinolone resistance genes in *C. stellaris* samples: *qnrB* and *qepA*.

The *qnrB* gene exhibited a particularly high prevalence, being present in 81 % of the lichen samples. It showed significantly higher relative abundance in southern lichens, which could be consistent with introductions via bioaerosol dispersal (Pilote *et al*., 2019; George *et al*., 2022) from anthropogenically influenced areas such as nearby agricultural and urban centres. However, this remains speculative and cannot be directly tested with our current dataset. Network analysis revealed positive correlations between *qnrB* and several bacterial genera, including *Connexibacter*, *Granulicella* and *Novosphingobium*. Interestingly, *Connexibacter*, which is known for its resistance to quinolones (Monciardini *et al*., 2003), showed the strongest correlation with *qnrB*. While this does not provide evidence that *Connexibacter* harbours the gene, it reflects a pattern of co-variation within the lichen-associated microbial community, possibly driven by shared ecological or selective pressures. *Novosphingobium* also showed a weaker but positive correlation with *qnrB*, consistent with previous reports linking *Novosphingobium* to other *qnr* genes (Yan *et al*., 2017).

Both *qnrB* and *blaCTX-M-1* were detected together in 77 % of the *C. stellaris* samples, but our network analysis did not reveal significant co-occurrence between the two genes. Previous studies have reported their joint presence in MGEs, such as *IncN* plasmids flanked by the MGE *IS26* (Juraschek *et al*., 2022), suggesting that these genes can be mobilized together. A similar mechanism could potentially contribute to their frequent co-detection in *C. stellaris*, but our data do not provide direct evidence to support this hypothesis.

The *qepA* gene, encoding a quinolone efflux pump, was detected in 57 % of the *C. stellaris* samples, with no significant differences in relative abundance between northern and southern LWs. Notably, *qepA* exhibited high relative abundance, often exceeding that of the 16S rRNA gene. This pattern is striking, since 16S rRNA genes typically occur in multiple copies per bacterial genome (ranging from 1 to >10; Louca *et al*., 2018; Pan *et al*., 2023), whereas *qepA* is usually found as a single copy on plasmids or other MGEs. The unusually high abundance of *qepA* could reflect (1) inflation due to plasmid multicopy replication, (2) the persistence of extracellular DNA containing *qepA* in the lichen thalli or (3) a genuine ecological enrichment, where bacteria carrying *qepA* are selectively favoured within the lichen microbiome. Our data do not allow discrimination among these alternatives. The third explanation, ecological enrichment, is consistent with the known roles of efflux pumps, which serve diverse physiological roles for bacteria, particularly detoxification of potentially harmful compounds (Piddock, 2006; Martínez *et al*., 2009; García-León *et al*., 2014). For instance, Alonso *et al*. (1999) demonstrated that environmental isolates of *Pseudomonas aeruginosa* collected prior to the widespread use of synthetic antibiotics could already expel quinolones, suggesting that these mechanisms originally evolved as defences against naturally occurring compounds. In the context of lichen symbioses, efflux pumps may therefore provide a key advantage by protecting bacteria against the diverse array of antimicrobial metabolites occurring in the environment, including those produced by lichens themselves (Ranković *et al*., 2007; Studzińska-Sroka *et al*., 2015; Taylor *et al*., 2023). In this framework, *C. stellaris* provides a relevant case study. While there is currently no evidence that this species synthesizes quinolone-type compounds, its diverse secondary metabolite profile could nonetheless favour the retention of versatile detoxification mechanisms such as *qepA*, potentially explaining both its prevalence and elevated abundance in *C. stellaris*-associated bacteria. This interpretation remains plausible given the ecological functions of efflux pumps, but it remains speculative in the absence of transcriptional, functional and genomic evidence linking *qepA* activity to lichen-associated selective pressures.

Network analysis revealed positive correlations between *qepA* and the genera *Tundrisphaera* and *Terriglobus*. These correlations do not provide direct evidence that these genera carry *qepA*, but they indicate that the relative abundances of these taxa co-vary with the abundance of this efflux pump gene across *C. stellaris* samples. This pattern may reflect ecological co-variation, where both the gene and these genera thrive under similar environmental conditions within the lichen microbiome.

### Occasional ARGs: aminoglycosides, sulfonamides and *int1-a-marko*

In addition to the dominant ARGs discussed above, we detected the occasional presence of other resistance genes and the integron marker gene *int1-a-marko* in *C. stellaris*. Specifically, the aminoglycoside resistance genes *aac(6’)-Ib* and *aac(3’)*, the sulfonamide resistance gene *sul1*, and the integron marker gene *int1-a-marko* were identified at low frequencies, predominantly in samples from the southern LW. Aminoglycosides are natural compounds produced by soil bacteria (Schatz *et al*., 1944), and resistance to these compounds is widespread across diverse natural environments (Li *et al*., 2015; Yan *et al*., 2017; Zhuang *et al*., 2021; Urban-Chmiel *et al*., 2022). In contrast, sulfonamides are synthetic antibiotics first introduced in the 1930s, widely used in both human and veterinary medicine (Landers *et al*., 2012), with resistance genes now also broadly disseminated in the environment (Nunes *et al*., 2020). The geographical clustering of these ARGs in southern LW samples is consistent with a possible influence from nearby urban areas, potentially mediated by bioaerosol dispersal. However, the very limited number of detections make it difficult to support this hypothesis, and alternative explanations, such as co-evolution, cannot be excluded. Additionally, we detected *sul2* in more than 40 % of samples from both northern and southern regions. The frequent detection of *sul2* may reflect widespread resistance. Sulfonamide resistance genes have been documented in bioaerosols (Zhang *et al*., 2018; Han and Yoo, 2020; Qu *et al*., 2024). However, they has also been detected in forest soils with no history of exposure to these synthetic drugs (Willms *et al*., 2019). These observations suggest that, although sulfonamides may not naturally occur in these environments, the genetic mechanisms for resistance can exist within microbial communities. Such persistence may indicate an adaptive capacity explaining *sul2* persistence within *C. stellaris*, despite limited exposure to antibiotics from anthropogenic uses. Nevertheless, our observations do not provide direct evidence on the factors driving *sul2* maintenance in *C. stellaris*, and targeted studies will be required to elucidate its origin and ecological role.

## CONCLUSION

To our knowledge, this is the first study to directly quantify and confirm the presence of ARGs in lichens using HT-qPCR. We detected ten ARGs and one MGE in the lichen *C. stellaris* from northern and southern LWs in eastern Canada, with a predominance of beta-lactam and quinolone resistance genes. Our results revealed differences in ARG relative abundances between northern and southern sites, although only *qnrB* showed significant variation. Furthermore, the bacterial community composition, while distinct between LWs, did not appear to drive ARG distribution.

Interpretations of ARG–taxon relationships should be viewed with caution, as they are based solely on co-occurrence patterns from 16S rRNA data and cannot establish direct host carriage. Future studies combining bacterial isolation with whole-genome sequencing or applying genome-resolved metagenomics (including metagenome-assembled genomes (MAGs) reconstruction and plasmid-focused assemblies) will be essential to determine the genomic context of the detected ARGs and to validate potential host–gene associations.

Similarly, our DNA-based approach establishes the presence of ARGs but does not reveal whether these genes are functionally active or represent extracellular or relic DNA persisting in the lichen thalli. DNA-based detection alone cannot distinguish between relic DNA and active transcription, which can only be demonstrated using RNA-based approaches such as reverse transcription quantitative PCR (RT-qPCR) or metatranscriptomics. Establishing whether the detected ARGs are expressed *in situ*, or instead represent non-functional remnants of past genetic events, will be essential to clarify their ecological relevance within the lichen microbiome.

Among the ARGs identified, we hypothesize that *blaCTX-M-1* and *qepA* may reflect ancient co-evolutionary processes within lichen-associated microbiomes, whereas the higher relative abundance of *qnrB* in southern samples may suggest more recent introductions influenced by anthropogenic activity. These scenarios remain speculative and require targeted validation. Future studies applying air sampling and atmospheric monitoring, chemical profiling of *C. stellaris* metabolites combined with antimicrobial susceptibility assays, and comparative analyses of co-occurring across lichens and soils will be critical to disentangle long-distance dispersal from endogenous selective processes.

Taken together, our results demonstrate that ARGs occur in *C. stellaris* across both northern and southern boreal lichen woodlands, including remote sites with limited direct human influence. This exploratory study establishes a framework for characterizing the lichen resistome and highlights key avenues for future research. As antibiotic resistance continues to pose a major global health challenge, understanding how resistance genes persist and spread even in isolated environments such as LWs represents an open and relevant scientific question.

## Supplementary Material

mcaf231_Supplementary_Data

## Data Availability

The 16S rRNA sequencing data analysed in this study were generated in a previous project and are publicly available in the NCBI Sequence Read Archive under BioProject accession number PRJNA593044. Detailed sample metadata, including BioSample accession numbers, geographical coordinates and collection details, are provided in Supplementary Data Table S1. The R script used for the analyses is publicly available at Zenodo: https://doi.org/10.5281/zenodo.16967960

## SUPPLEMENTARY DATA

Supplementary data are available at *Annals of Botany* online and consist of the following. Table S1. Detailed information on the *Cladonia stellaris* samples analysed in this study and collected from northern lichen woodlands in Kuujjuarapik–Whapmagoostui and southern lichen woodlands in Parc National des Grands-Jardins (PNGJ). Each entry includes the voucher number, collector’s name, collection number, geographical position, specific collection sites, altitude range, and precise latitude and longitude coordinates. Additionally, for each sample, the BioSample accession number (Accession) and associated BioProject code (BioProject) registered in the NCBI database are provided. Table S2. List of bacterial genera identified in *Cladonia stellaris* samples with their respective ASV counts. Table S3. Relative abundance of antibiotic resistance genes (ARGs) and a mobile genetic element (MGE) in *Cladonia stellaris* from northern and southern lichen woodlands (LWs). The columns list the specific ARGs detected, and the rows represent individual lichen samples. The LW from which each sample originates is indicated. Numerical values indicate the abundance of each ARG/MGE relative to the 16S rRNA gene, with values greater than 1 signifying a higher abundance than 16S rRNA, and values less than 1 indicating a lower abundance. ‘NA’ denotes that the gene was not detected in the corresponding sample. Table S4. Summary of network metrics for bacterial genera and antibiotic resistance genes (ARGs) in *Cladonia stellaris* from northern and southern lichen woodlands (LWs). The table presents detailed network statistics, including degree, closeness centrality, betweenness centrality, clustering coefficient, average shortest path length, eccentricity, neighbourhood connectivity, number of directed and undirected edges, stress, radiality and topological coefficient. Metrics are shown for all samples. For bacterial genera, additional columns include the phylum, class, order, family and genus information, while ARGs are listed with their specific resistance mechanisms. When the genus is unknown, the bacteria are identified by the family name followed by ‘Unclassified’. Figure S1. Relative abundance per phylum (A) and genera (B) of bacteria associated with *Cladonia stellaris*. Samples are grouped by latitude of lichen woodland (LW), northern or southern, as indicated at the top of the bar plot. Each vertical bar represents a single sample, and colours reflect different phyla (A) or genera (B). In B, unassigned taxa were removed prior to plotting, which may result in bar heights summing to less than one. Figure S2. Total relative abundance of ten antibiotic resistance genes (ARGs) and a mobile genetic element (MGE) in *Cladonia stellaris* from northern and southern lichen woodlands (LWs). The boxplots depict the median (line in the box), quartiles (edges of the box) and range of values within 1.5 times the interquartile range (whiskers) of the 11 ARGs/MGE within each LW. Individual points represent individual observations. The *y*-axis is on a logarithmic scale to better visualize differences. Wilcoxon rank-sum results indicate no significant differences between the northern and southern LWs (adjusted *P* = 0.3669).
