# Supplementary material for: Antibiotic resistance genes detected in lichens: insights from Cladonia stellaris
Source: Ann Bot. 2025 Sep 22;137(1):233–46. doi: 10.1093/aob/mcaf231 (PMC12784081; doi:10.1093/aob/mcaf231)
Supplement: mcaf231_Supplementary_Data [file mcaf231_supplementary_data.zip › FigureS01.pdf]

Antibiotic resistance genes detected in lichens: insights from *Cladonia stellaris*

Marta Alonso-García, Paul B. L, George, Samantha Leclerc, Marc Veillette, Caroline Duchaine and Juan Carlos Villarreal A.

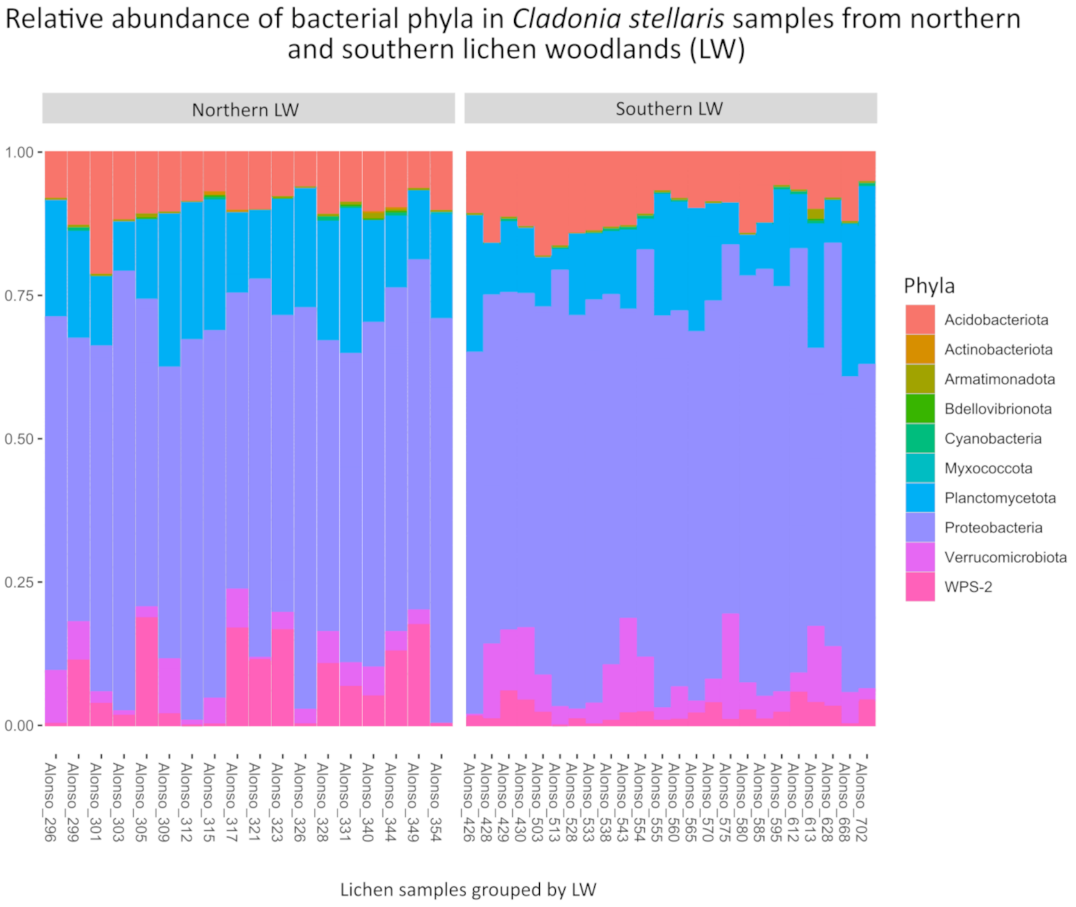

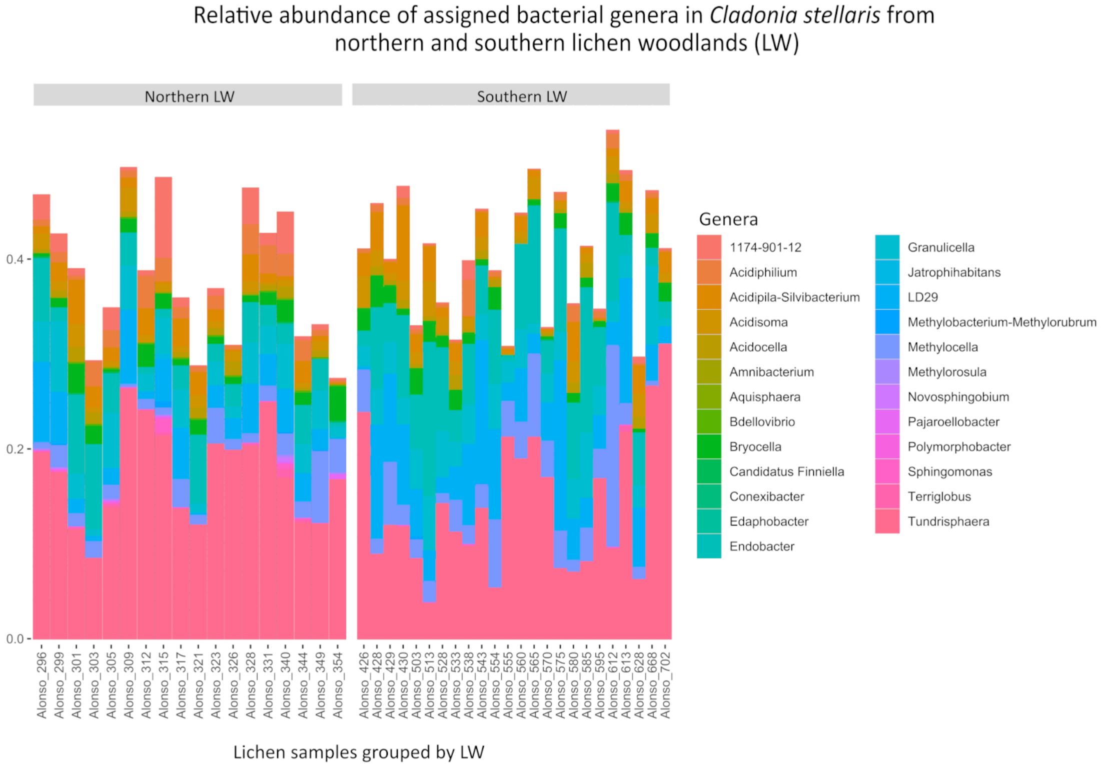

Figure S1. Relative abundance per phylum (A) and genera (B) of bacteria associated to Cladonia stellaris. Samples are grouped by latitude of lichen woodland (LW), northern or southern, as indicated at the top of the bar plot. Each vertical bar represents a single sample, and colors reflect different phyla (A) or genera (B). In panel B, unassigned taxa were removed prior to plotting, which may result in bar heights summing to less than one.

Annals of Botany - https://doi.org/10.1093/aob/mcaf231
