# Supplementary material for: Antibiotic resistance genes detected in lichens: insights from Cladonia stellaris
Source: Ann Bot. 2025 Sep 22;137(1):233–46. doi: 10.1093/aob/mcaf231 (PMC12784081; doi:10.1093/aob/mcaf231)
Supplement: mcaf231_Supplementary_Data [file mcaf231_supplementary_data.zip › FigureS02.pdf]

Antibiotic resistance genes detected in lichens: insights from *Cladonia stellaris*

Marta Alonso-García, Paul B. L, George, Samantha Leclerc, Marc Veillette, Caroline Duchaine and Juan Carlos Villarreal A.

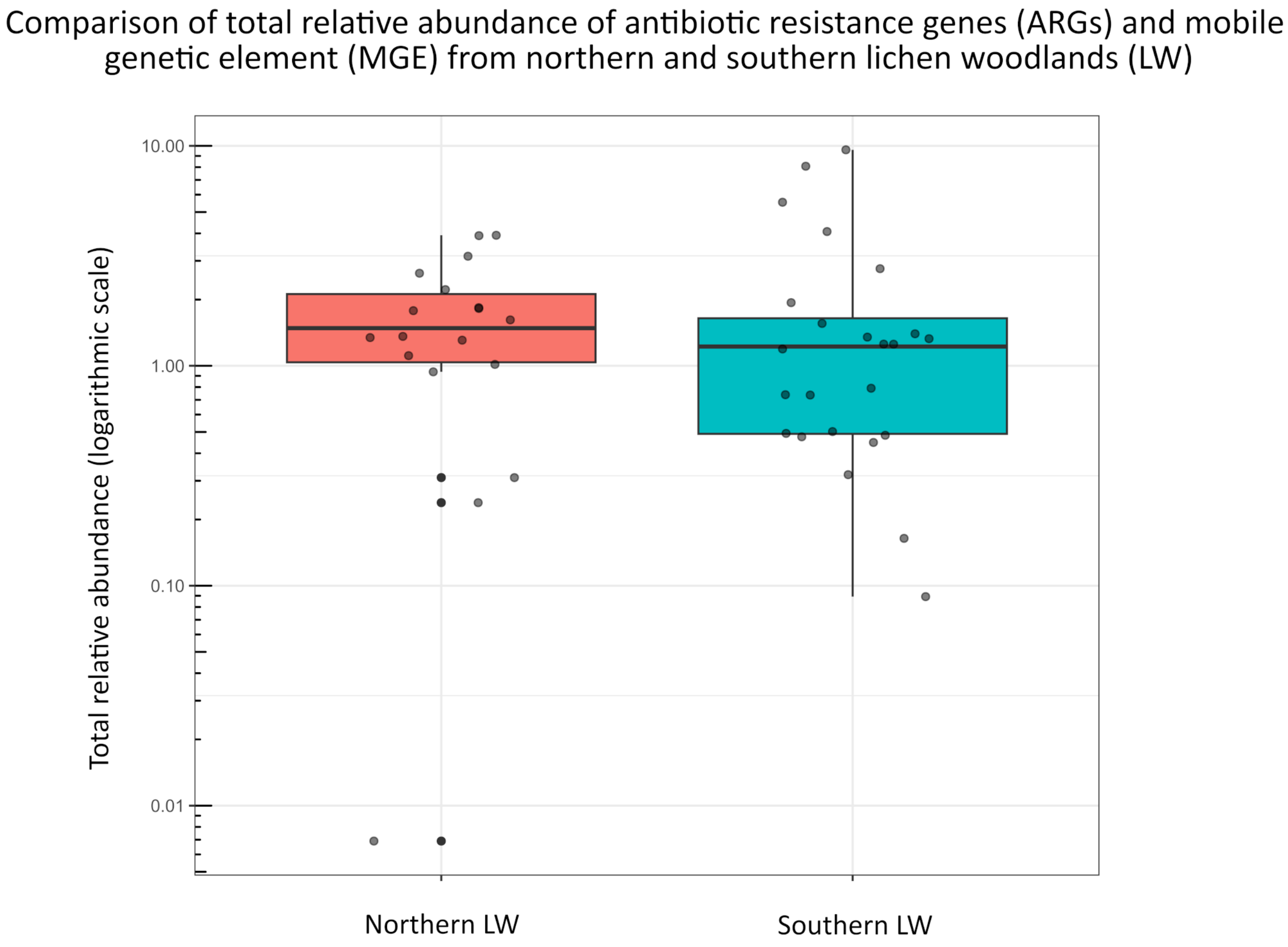

Figure S2. Total relative abundance of ten antibiotic resistance genes (ARGs) and a mobile genetic element (MGE) in Cladonia stellaris from northern and southern lichen woodlands (LWs). The boxplots depict the median (line in the box), quartiles (edges of the box), and range of values within 1.5 times the interquartile range (whiskers) of the 11 ARGs/MGE within each LW. Individual points represent individual observations. The y-axis is on a logarithmic scale to better visualize differences. Wilcoxon rank-sum results indicate no significant differences between the northern and southern LWs (adjusted p-value = 0.3669)..
